# Normative annual changes in left ventricular volumes, mass and function among healthy adults: a prospective cardiac magnetic resonance imaging study

**DOI:** 10.1186/1532-429X-16-S1-P99

**Published:** 2014-01-16

**Authors:** William E Moody, Nicola C Edwards, Colin D Chue, Robin J Taylor, Andrew You Kuan Li, Charles J Ferro, John N Townend, Richard P Steeds

**Affiliations:** 1 Birmingham Cardio-Renal Group, University of Birmingham, Birmingham, UK; 2 Cardiology, Queen Elizabeth Hospital Birmingham, Birmingham, UK; 3 Renal, Queen Elizabeth Hospital Birmingham, Birmingham, UK

## Background

Cardiac magnetic resonance imaging (CMR) is routinely performed to monitor progression and response to interventions in congenital and acquired heart diseases. Cross-sectional CMR studies report normalized reference ranges for cardiac parameters according to age decile together with assessments of short-term inter-study variability; however, most clinical imaging is repeated at much greater intervals of time. There are no prospective CMR data defining long-term changes in cardiac structure and function among healthy subjects. Without such data, it is difficult to determine whether serial changes in cardiac parameters observed in patients reflect disease progression or are merely the consequence of normal ageing. We examined the "within patient" changes in left ventricular (LV) volumes, mass and function over 12 months in a well-characterized cohort of healthy adults.

## Methods

We identified 21 healthy controls (age 39 ± 13 yr, male 71%) who did not proceed to uni-nephrectomy after recruitment into an observational study assessing the cardiovascular effects of live kidney donation (NCT01028703). All subjects were asymptomatic and had a 10-year risk of a cardiovascular event of <20% (QRISK-2), a normal stress echocardiogram and normal haematology and biochemistry profiles. Exclusion criteria included: any history of cardiovascular disease, including hypertension; diabetes; glucose intolerance; chronic kidney disease; first degree relative with a proven or potentially inheritable cardiac condition. Subjects underwent CMR imaging (1.5T Magnetom Avanto, Siemens) at baseline and 12 months. Analysis of SSFP images was performed offline (Argus Software, Siemens) by a single blinded observer (W.E.M.) for assessment of LV volumes, systolic function and mass. Baseline studies were repeat analyzed by the same trained observer to assess intra-observer variability. A subset of participants underwent repeat imaging within 1 hour to determine inter-study variability.

## Results

There were no significant changes in any LV parameter indexed to body surface area at 12 months (Table 1). There was however, a borderline significant reduction in absolute LV mass (p = 0.04). This finding is supported by existing CMR data which describe reductions in LV mass associated with advancing age. It may in part also reflect the "Hawthorne effect", whereby participants adopt healthier lifestyle behaviours in reaction to being observed. For all other LV parameters, short-term inter-study biases were comparable to any mean differences observed at 12 months (Tables 1 and 2).

**Table 1:** Annual change in left ventricular volumes, mass and function in healthy adult subjects

Parameter	Month 0	Month 12	Mean difference	p
LVEF (%)	69.4 ± 7.8	70.4 ± 6.8	1.1 ± 3.5	0.25

LVEDV (mL)	136.0 ± 27.8	136.3 ± 23.4	0.3 ± 10.9	0.91

LVEDVI (mL/m2)	70.2 ± 16.5	70.2 ± 13.2	-0.03 ± 6.0	0.99

LVESV (mL)	43.3 ± 17.2	41.3 ± 14.1	-2.0 ± 6.8	0.26

LVESVI (mL/m2)	22.3 ± 9.5	21.3 ± 7.2	-1.0 ± 3.8	0.29

LVSV (mL)	92.9 ± 13.7	95.1 ± 13.1	2.1 ± 8.3	0.32

LVSVI (mL/m2)	47.8 ± 8.6	48.9 ± 7.9	1.1 ± 4.6	0.37

LVM (g)	131.5 ± 27.6	126.8 ± 27.8	-4.8 ± 8.4	0.04*

LVMI (g/m2)	67.5 ± 14.5	65.1 ± 13.5	-2.4 ± 5.0	0.07

Data are mean ± SD. Changes in LV parameters are compared using a paired Student's t test. LVEF, left ventricular ejection fraction; LVEDV, left ventricular end diastolic volume; LVEDVI, left ventricular end diastolic volume index; LVESV, left ventricular end systolic volume; LVESVI, left ventricular end systolic volume index; LVSV, left ventricular stroke volume; LVSVI, left ventricular stroke volume index; LVM, left ventricular mass; LVMI, left ventricular mass index

**Table 2:** Intra-observer and short-term inter-study variability for left ventricular volumes, mass and function in healthy adult subjects

Parameter	Variability	Bias ± SD	p	LoA	CV (%)	ICC (95% CI)
LVEF (%)	Intra-observer Inter-study	1.00 ± 1.27 1.50 ± 1.76	0.11 0.09	-1.48 to 3.48 -1.95 to 4.95	1.8 2.4	0.99 (0.94 to 1.00) 0.98 (0.89 to 1.00)

LVEDV (mL)	Intra-observer Inter-study	0.67 ± 3.62 0.50 ± 6.47	0.67 0.86	-6.43 to 7.77 -12.18 to 13.18	2.6 4.7	0.99 (0.95 to 1.00) 0.98 (0.85 to 1.00)

LVESV (mL)	Intra-observer Inter-study	-0.83 ± 2.79 -1.33 ± 5.00	0.50 0.54	-6.30 to 4.64 -11.13 to 8.47	7.0 12.5	0.99 (0.93 to 1.00) 0.97 (0.78 to 1.00)

LVSV (mL)	Intra-observer Inter-study	1.00 ± 2.97 1.67 ± 4.03	0.45 0.36	-4.82 to 6.82 -6.23 to 9.57	3.1 4.2	0.98 (0.85 to 1.00) 0.96 (0.74 to 0.99)

LVM (g)	Intra-observer Inter-study	-0.33 ± 2.16 0.00 ± 3.03	0.21 1.00	-4.56 to 3.90 -5.94 to 5.94	1.5 2.1	0.99 (0.95 to 1.00) 0.99 (0.90 to 1.00)

CV, coefficient of variability; ICC, intra-class correlation coefficient; LoA, limits of agreement; LVEF, left ventricular ejection fraction; LVEDV, left ventricular end diastolic volume; LVESV, left ventricular end systolic volume; LVSV, left ventricular stroke volume; LVM, left ventricular mass.

## Conclusions

These data have particular importance for both clinical practice and observational research: for patients undergoing annual CMR, changes in LV structure and function can be largely attributed to disease progression rather than ageing.

## Funding

WEM is supported by a British Heart Foundation Clinical Research Training Fellowship.

